# Machine Learning–Based Approach for Identifying Research Gaps: COVID-19 as a Case Study

**DOI:** 10.2196/49411

**Published:** 2024-03-05

**Authors:** Alaa Abd-alrazaq, Abdulqadir J Nashwan, Zubair Shah, Ahmad Abujaber, Dari Alhuwail, Jens Schneider, Rawan AlSaad, Hazrat Ali, Waleed Alomoush, Arfan Ahmed, Sarah Aziz

**Affiliations:** 1 AI Center for Precision Health Weill Cornell Medicine-Qatar Doha Qatar; 2 Department of Nursing Hamad Medical Corporation Doha Qatar; 3 Division of Information and Computing Technology College of Science and Engineering Hamad Bin Khalifa University Doha Qatar; 4 Nursing Department Hamad Medical Corporation Doha Qatar; 5 Information Science Department College of Life Sciences Kuwait University Kuwait Kuwait; 6 Health Informatics Unit Dasman Diabetes Institute Kuwait Kuwait; 7 Faculty of Computing and Information Technology Sohar University Sohar Oman; 8 School of Information Technology Skyline University College Sharjah United Arab Emirates

**Keywords:** research gaps, research gap, research topic, research topics, scientific literature, literature review, machine learning, COVID-19, BERTopic, topic clustering, text analysis, BERT, NLP, natural language processing, review methods, review methodology, SARS-CoV-2, coronavirus, COVID

## Abstract

**Background:**

Research gaps refer to unanswered questions in the existing body of knowledge, either due to a lack of studies or inconclusive results. Research gaps are essential starting points and motivation in scientific research. Traditional methods for identifying research gaps, such as literature reviews and expert opinions, can be time consuming, labor intensive, and prone to bias. They may also fall short when dealing with rapidly evolving or time-sensitive subjects. Thus, innovative scalable approaches are needed to identify research gaps, systematically assess the literature, and prioritize areas for further study in the topic of interest.

**Objective:**

In this paper, we propose a machine learning–based approach for identifying research gaps through the analysis of scientific literature. We used the COVID-19 pandemic as a case study.

**Methods:**

We conducted an analysis to identify research gaps in COVID-19 literature using the COVID-19 Open Research (CORD-19) data set, which comprises 1,121,433 papers related to the COVID-19 pandemic. Our approach is based on the BERTopic topic modeling technique, which leverages transformers and class-based term frequency-inverse document frequency to create dense clusters allowing for easily interpretable topics. Our BERTopic-based approach involves 3 stages: embedding documents, clustering documents (dimension reduction and clustering), and representing topics (generating candidates and maximizing candidate relevance).

**Results:**

After applying the study selection criteria, we included 33,206 abstracts in the analysis of this study. The final list of research gaps identified 21 different areas, which were grouped into 6 principal topics. These topics were: “virus of COVID-19,” “risk factors of COVID-19,” “prevention of COVID-19,” “treatment of COVID-19,” “health care delivery during COVID-19,” “and impact of COVID-19.” The most prominent topic, observed in over half of the analyzed studies, was “the impact of COVID-19.”

**Conclusions:**

The proposed machine learning–based approach has the potential to identify research gaps in scientific literature. This study is not intended to replace individual literature research within a selected topic. Instead, it can serve as a guide to formulate precise literature search queries in specific areas associated with research questions that previous publications have earmarked for future exploration. Future research should leverage an up-to-date list of studies that are retrieved from the most common databases in the target area. When feasible, full texts or, at minimum, discussion sections should be analyzed rather than limiting their analysis to abstracts. Furthermore, future studies could evaluate more efficient modeling algorithms, especially those combining topic modeling with statistical uncertainty quantification, such as conformal prediction.

## Introduction

### Background

Scientific research relies on applying systematic scientific methods and actions to increase knowledge in fields or specific topics [1]. One essential first step in engaging in scientific research is identifying research gaps [2], where insufficient data, knowledge, or understanding limit our ability to draw conclusions in a given field or topic [3]. Research gaps can also be referred to as unanswered questions that have not yet been addressed or are underexplored in the existing body of knowledge, either due to a lack of studies or inconclusive results [4,5]. Research gaps can also serve as a starting point for research as well as motivate further research [6]. Researchers have classified research gaps into seven categories [3,6,7]: (1) evidence gaps—where contradictions in the findings of the previous research exist; (2) knowledge gaps—where knowledge may either not exist in the literature or the results deviated from what was expected; (3) practical-knowledge conflict gaps—where the goal is to discover the reasons and scope of differences between professionals’ behaviors versus their advocated behavior; (4) empirical gaps—where there is a need to empirically evaluate and verify research findings or propositions; (5) methodological gaps—where shortcomings may arise due to having a single methodology influencing the research results; (6) theoretical gaps—related to examining gaps that exist in theories and their models and compare them with prior research; and (7) population gaps—where a population is not adequately represented or underresearched in prior studies.

The sheer volume and accelerated pace of scientific research output present both opportunities and challenges. Identifying potential interventions, best practices, and policy recommendations, all backed up by evidence is made easier by the wealth of information available. However, researchers face challenges with staying up-to-date with the latest findings, identifying redundancies in research, and objectively identifying research gaps that need to be addressed. Therefore, it becomes paramount to accurately identify the research gaps to advance our understanding of the issue or topic, better use the allocation of resources, and better inform evidence-based policy making.

Traditionally, several methods are used to identify research gaps, including literature reviews, systematic reviews, expert opinions, and consensus-building activities (eg, developing guidelines). Yet, such methods require an intensive time commitment, are prone to bias, and can be labor intensive. Additionally, such methods may not be suitable to address issues, where the evidence or research subject is rapidly increasing in volume and pace or is time sensitive (eg, COVID-19). Consequently, there is a need for innovative and scalable approaches to systematically assess existing literature, identify research gaps, and prioritize areas for further study in the topic or field of interest.

Machine learning (ML) techniques have demonstrated great potential applications of scientific insights and discoveries by addressing challenges related to information retrieval, knowledge discovery, and natural language processing [8]. ML is a branch of computational science and a subset of artificial intelligence (AI); it focuses on the development of algorithms that enable machines to learn from [9-12] and make predictions or decisions based on data, without being directly programmed [13]. Broadly, ML algorithms can “learn” through supervised learning, unsupervised learning (eg, reinforcement learning), or a mixture thereof, referred to as semisupervised learning [14,15].

### Research Problem and Aim

In the context of identifying research gaps, ML techniques may facilitate the discovery of research gaps by analyzing large volumes of scientific evidence in a systematic, scalable, and efficient manner. To understand the current status quo of scientific evidence available, several studies leveraged ML to perform natural language processing, bibliometric analysis, and text mining, which have yielded promising results in several domains, including health care [9-12], social sciences [16-19], and environmental sciences [20]. However, the application of ML and its potential for identifying research gaps in rapidly evolving fields remains underexplored. To the best of our knowledge, there are no previous studies that leveraged ML-based techniques for identifying research gaps.

This paper aims to propose an ML-based approach to detect research gaps in the literature. In this work, we use the novel COVID-19 pandemic, caused by the SARS-CoV-2 virus, as a case study due to the fast and time-critical pace it evolved, along with the urgent need to comprehend this global pandemic. Since its emergence in late 2019, COVID-19 has had a profound global impact which not only halted many activities, triggered economic fallouts, and caused significant hardships, but it also claimed more than 6.8 million lives. In turn, the scientific community united to address the various challenges presented by the global spread of the virus.

Through unprecedented levels of scientific activities, researchers generated a vast amount of research output, with thousands of research papers being published each month, which discuss various aspects of the virus, its transmission, diagnosis, treatment, and prevention strategies [9,21,22]. However, with the rapid pace of information generation and dissemination, there is an increased risk of overlooking research gaps or underexplored areas that may be critical to understanding and mitigating the virus’s impact. Thus, this aims to propose an ML-based approach to detect research gaps in the literature using COVID-19 as a case study.

## Methods

### Study Data Selection

We used the COVID-19 Open Research (CORD-19) data set, produced by the Allen Institute for AI and made available on the Kaggle platform [23]. It was updated every week until June 2, 2022, to include the most recently published COVID-19 papers. We used the final version, which contains 1,121,433 entries. The search terms used by Allen Institute for AI to retrieve these studies were “Coronavirus” OR “Corona virus” OR “COVID-19” OR “2019-nCoV” OR “SARS-CoV” OR “Severe Acute Respiratory Syndrome” OR “MERS-CoV” OR “Middle East Respiratory Syndrome” [24]. The sources of studies in the CORD-19 data set were PubMed Central, PubMed, the World Health Organization’s (WHO) COVID-19 Database, arXiv, bioRxiv, and medRxiv [24]. The data set includes a CSV file containing meta-information about all the papers, such as article DOI, CORD UID, PMCID, PUBMED ID, title, abstract, journal, authors, and publication date. We removed duplicate papers, entries with empty and non-English abstracts, and papers published before January 1, 2020. We selected abstracts containing the keywords “novel coronavirus,” “coronavirus 2019,” “2019-nCov,” “COVID-19,” “COVID 2019,” “severe acute respiratory syndrome coronavirus 2,” and “SARS-COV-2” to include only COVID-19–related papers.

### Data Preprocessing

We cleaned the abstracts and removed nonalphanumeric characters, punctuations, and sectioning keywords “BACKGROUND,” “OBJECTIVE,” “METHOD,” “RESULT,” “CONCLUSION.” We used the Python programming language in a Jupyter Notebook environment and Python libraries such as *pandas*, *NumPy*, *langdetect*, *re*, *string*, and *TextBlob*. We then searched abstracts that mentioned any term related to research gap: “unknown,” “not known,” “little is known,” “unrevealed,” “uncertain,” “undetermined,” “understudied,” “unexplored,” “not fully understood,” “literature gap,” “research gap,” “knowledge gap,” “future studies,” “future research,” “research problem,” “more studies,” “more research,” “further studies,” and “further research.” We decided to use these terms in an abstract search rather than a full-text search for 2 reasons. First, using these terms in a full-text search may lead to the inclusion of a significant number of unrelated studies, increasing the likelihood of inaccurately identifying research gaps that are not pertinent to the subject of interest. Second, the CORD-19 data set that we used in this study does not contain the full text of the studies. With this process, we identified 33,206 abstracts of scholarly papers published after January 1, 2020, related to COVID-19 and containing the gap words. We analyzed these abstracts, and from each abstract, we extracted 3 sentences: 1 sentence that includes the gap word, 1 sentence before, and 1 sentence after the sentence containing the gap word. We used a full stop (“.”) as a sentence marker. We converted the selected sentences to lowercase text. Next, we used the Python NLTK library to remove the stop words and tokenize the sentences. Finally, we used the clean sentences after the preprocessing steps for clustering.

### Analysis

#### Overview

**Figure 1:**
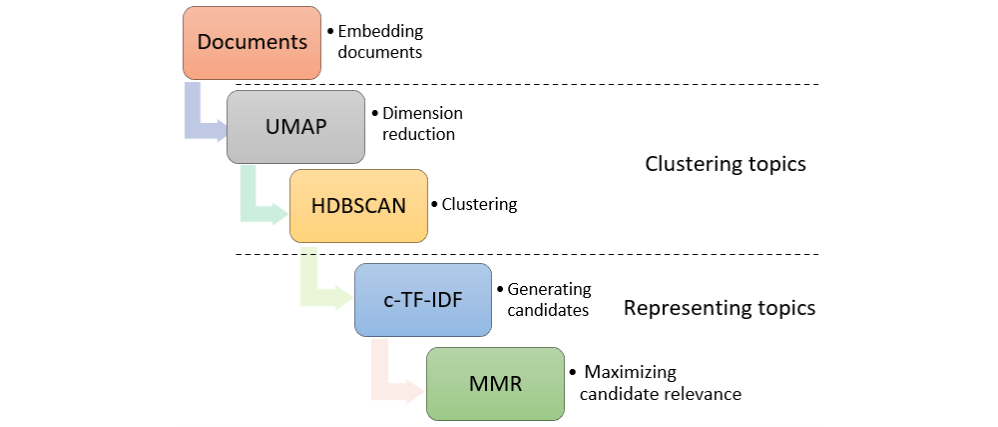
Stages of the BerTopic modeling algorithm. c-TF-IDF: class-based term frequency-inverse document frequency; MMR: maximum marginal relevance; HDBSCAN: hierarchical density-based spatial clustering of applications with noise; UMAP: uniform manifold approximation and projection.

For clustering the sentences into semantically similar topics, we used the BERTopic algorithm [25]. The BERTopic algorithm is an unsupervised learning algorithm for topic modeling. It uses the Bidirectional Encoder Representations from Transformers (BERT). BERTopic does not require labeled data as it extracts topics from an input text in a supervised way [26]. BERTopic gained popularity due to its potential to capture context-aware information in a given input text and does not rely on a predefined number of topics [26]. Besides topic modeling, BERTopic is also useful in the clustering of documents and text summarization [26]. The BERTopic topic modeling method produces dense clusters by combining class-based term frequency-inverse document frequency (TF-IDF) and transformers (BERT embeddings). In addition, it makes it simple to comprehend and visualize the generated topics. The 3 stages in the BERTopic algorithm are presented in Figure 1 and discussed in the next subsections.

#### Embedding Documents

In this stage, the BERTopic algorithm extracts document embeddings using BERT sentence transformers. Each document embedding is generated using word embeddings, which represent each word in the document in multidimensional space. It ensures that words with related meanings have comparable representations. This way, words are represented by numbers in a vector space, where vectors are defined by TF-IDF weights. TF-IDF describes the importance of a term relative to a document in a corpus. Neural networks are the primary foundation of embedding models. Typically, word embeddings are used to compute document embedding into 2 phases. The word embedding is first applied to every word in the text and then the word embeddings are aggregated by averaging over each dimension to produce document embeddings.

#### Clustering Documents

Documents embeddings produced in the previous step are usually very sparse. Therefore, our first stage of the analysis uses uniform manifold approximation and projection to decrease the dimensionality of the embeddings [27]. Then, we use the hierarchical density-based spatial clustering of applications with a noise approach to cluster reduced embeddings and produce clusters of texts with comparable semantic properties.

#### Representing Topics

In this stage, class-based TF-IDF weights are used to extract topics. These topics are reduced and further improved by finding the coherence of words using maximum marginal relevance. We used BERTopic to cluster the extracted sentences and found 191 clusters. Then, we applied the topics reduction technique of BERTopic and found a total of 50 clusters. We assigned labels to each cluster by checking their representative words. When it was challenging to assign labels to a cluster based on its representative words, we reviewed sentences containing the specified research gap terms, along with the sentence before and after in most studies within that cluster. After that, we merged clusters that had similar labels to identify 21 unique labels (research gaps). Finally, we grouped these clusters into 6 broader categories. Multimedia Appendix 1 shows the code used for the analysis of bibliographic data to identify research gaps.

## Results

### Search Results

**Figure 2:**
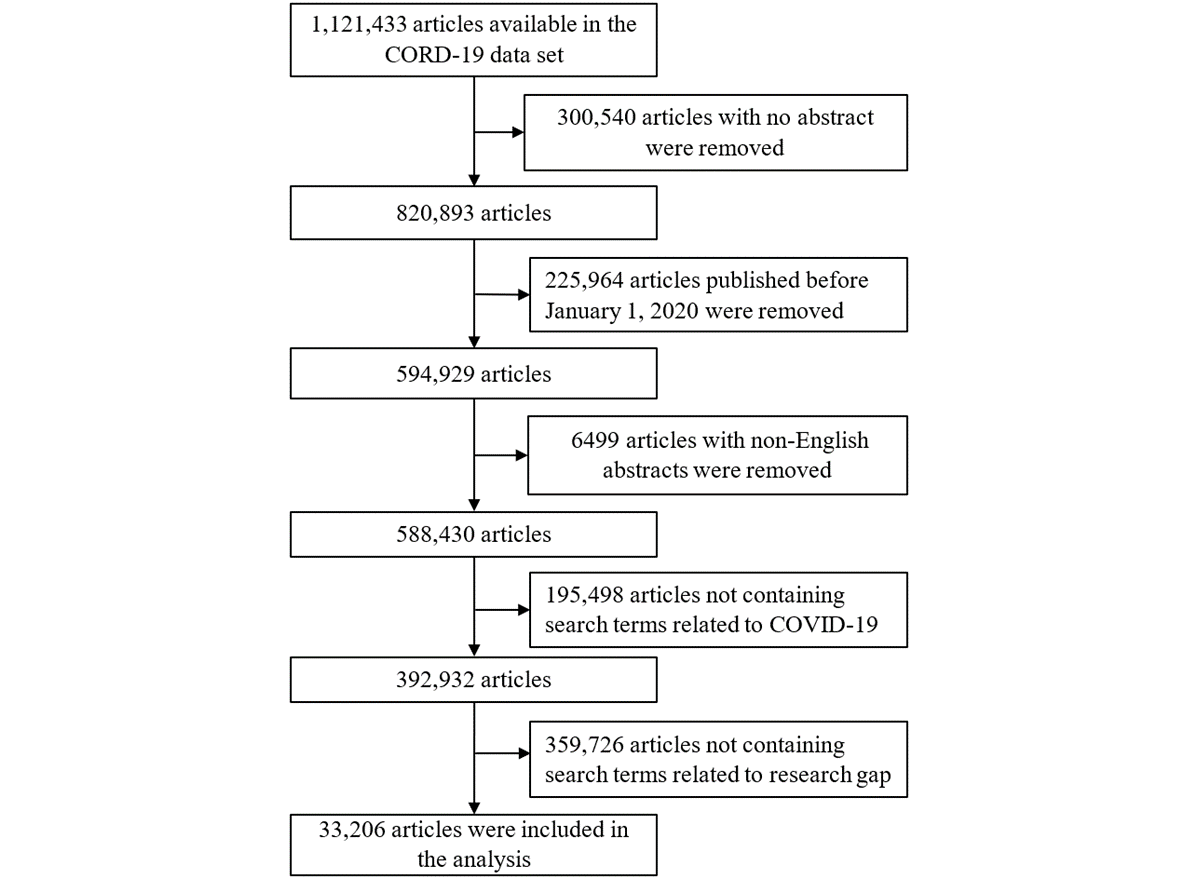
Flowchart of the selection of published articles. CORD-19: COVID-19 Open Research.

By June 2, 2022, the CORD-19 data set contained 1,121,433 papers (Figure 2). Of those, we excluded 1,088,227 papers for the following reasons: (1) abstracts were unavailable (n=300,540); (2) the papers were published before January 1, 2020 (n=225,964); (3) the papers were written in a language other than English (n=6499); (4) papers did not contain search terms related to COVID-19 (n=195,498); and (5) papers did not contain search terms related to research gap (n=359,726). Consequently, we included 33,206 papers in the analysis of this study.

### Results of Gap Identification

#### Overview

As mentioned earlier, 191 clusters of the 33,206 papers were generated by our analysis. Then, the number of clusters was reduced to 50 when we applied a topics reduction technique. We deleted 6 clusters as we could not identify the research gap from them. Further, 23 clusters were merged with other clusters as the same research gaps were identified in these clusters. Overall, we identified 21 different research gaps from 4646 papers. As shown in Table 1, these research gaps were grouped into 6 topics, which are discussed in the next subsections.

**Table 1:** Topics and the corresponding subtopics identified in this study (N=4646).

Topics and subtopics		Values, n (%)
**Topic 1: virus of COVID-19**		
	Origin of COVID-19	318 (6.8)
	Emerging variants	202 (4.3)
	Transmission of COVID-19	75 (1.6)
	Role of the immune system	52 (1.1)
**Topic 2: risk factors of COVID-19**		
	Obesity	77 (1.7)
	Ethnicity	65 (1.4)
**Topic 3: prevention of COVID-19**		
	Vaccination	447 (9.6)
	Precautionary measures	124 (2.7)
	Wastewater surveillance	60 (1.3)
**Topic 4: treatment of COVID-19**		
	Medications of COVID-19	170 (3.7)
	Herbal medicine	66 (1.4)
	Support system	55 (1.2)
**Topic 5: health care delivery during COVID-19**		
	Telehealth	305 (6.6)
	Surgeries	158 (3.4)
	Organ transplantation	92 (2)
**Topic 6: impact of COVID-19**		
	Health complications	1552 (33.4)
	Special groups	434 (9.3)
	Education	136 (2.9)
	Media and communication	109 (2.3)
	Mortality	87 (1.9)
	Food security	62 (1.3)

#### Topic 1: Virus of COVID-19 (n=647, 13.9%)

##### Origin of COVID-19

There are 318 studies related to the origin of COVID-19. Despite various theories and pieces of evidence, the exact source of SARS-CoV-2 remains unknown according to the majority of studies on this topic.

##### Emerging Variants

According to the 202 studies on this topic, the full extent of the impact of new variants on the course of the pandemic and the effectiveness of current prevention and treatment strategies on the new variants are still not known. Furthermore, it is uncertain how the emergence of new variants may affect the duration of immunity after recovery. In addition, it is not clear how accurate the prediction models are in predicting the emergence of new variants and their impact.

##### Transmission of COVID-19

A total of 75 studies have revealed several unknown aspects of the virus such as the role of asymptomatic carriers in the spread of the virus, the true level of global spread, and the extent to which COVID-19 may be circulating undetected in some areas, and the influence of environmental factors (eg, temperature and humidity) on the transmission of COVID-19.

##### Role of the Immune System

In 52 studies related to this topic, several research gaps were identified. Specifically, while it is known that the virus elicits an immune response, it is still unclear the specifics of how the immune system responds, the duration of immunity after recovery, the relationship between the severity of illness and the strength of the immune response, and how the immune response may differ between individuals.

#### Topic 2: Risk Factors of COVID-19 (n=142, 3.1%)

##### Obesity

From 77 studies related to this topic, we found that the relationship between obesity and COVID-19 is still largely a mystery. While some studies have suggested that obesity may increase the risk of severe COVID-19 outcomes, the exact mechanisms behind this are still unknown. Additionally, it is unclear whether weight loss can reduce the risk of severe illness and death from COVID-19 in individuals with obesity. Furthermore, there is no consensus on the optimal BMI cut-off for identifying individuals with obesity who are at increased risk of severe illness and death from COVID-19.

##### Ethnicity

A total of 65 studies identified a significant research gap in understanding the relationship between ethnicity and COVID-19. Despite the numerous studies conducted, the reasons for these disparities remain largely unknown.

#### Topic 3: Prevention of COVID-19 (n=631, 13.6%)

##### Vaccination

A total of 447 studies have revealed that the long-term efficacy and safety of COVID-19 vaccines remain largely unknown. Although initial clinical trials have demonstrated promising results, it is uncertain how long the protection provided by the vaccines will last and what potential long-term side effects may be. Further, vaccine hesitancy and the factors that contribute to it are not fully understood. Moreover, the safety of COVID-19 vaccines in patients with chronic diseases (eg, chronic hepatitis, diabetes, heart failure, renal failure, and epilepsy) remains unknown.

##### Precautionary Measures

A total of 124 studies were related to precautionary measures implemented to prevent the spread of the virus. According to these studies, the effectiveness of many precautionary measures such as social distancing, wearing masks, hand hygiene, respiratory etiquette (eg, covering mouth and nose when coughing and sneezing), and ventilation systems is still unknown. Further, the duration of quarantine or isolation for individuals with COVID-19 remains uncertain.

##### Wastewater Surveillance

In total, 60 studies were related to using wastewater surveillance to detect the presence of the virus in communities. According to these studies, it is unclear how accurately wastewater surveillance can predict or track COVID-19 outbreaks and what the potential false-positive or false-negative rates may be.

#### Topic 4: Treatment of COVID-19 (n=291, 6.3%)

##### Medications of COVID-19

The role of medications (eg, remedisvier and corticosteroids) in managing severe cases of COVID-19 was discussed in 170 studies. According to these studies, there are still many unknowns about the medication for COVID-19, including the potential for long-term effects on the heart, lungs, and other organs; the optimal treatment protocol (eg, timing, dose, and duration) for different stages of the disease and age groups; the effectiveness of existing medications against new variants and new medications against multiple variants; the safety and efficacy of a combination of medications; the best combinations and dosages; the safety and efficacy of medication in children; and the effect of the combination of medication and dietary supplements.

##### Herbal Medicine

Herbal medicine was the main topic in 66 studies. According to these studies, much remains unknown about the effectiveness, safety, standardization, dosage, and potential interactions with other treatment regimens.

##### Support System

A total of 55 studies were related to support systems that provide several services (eg, social support, mental health, and financial assistance) to individuals affected by COVID-19. According to these studies, the effectiveness and long-term impacts of support systems on patients with COVID-19 remain largely unknown.

#### Topic 5: Health Care Delivery During COVID-19 (n=555, 11.9%)

##### Telehealth

The COVID-19 pandemic has seen a dramatic surge in the use of telehealth; yet, a total of 305 studies have revealed that there are still many unknowns about its effectiveness and accessibility. These unknowns have the potential to create new privacy and security risks, as well as to impact health care disparities.

##### Surgeries

According to the 158 studies discussing this topic, there is still no consensus on planning to maintain surgical care preparedness in ongoing and future pandemics. This includes understanding the risk of transmission, perioperative testing criteria, postoperative outcomes in specific populations, and effective strategies for resource allocation.

##### Organ Transplantation

A total of 92 studies have revealed that there is still much to be discovered about the best way to manage organ transplantation during the COVID-19 pandemic. Specifically, there is still much to be uncovered about COVID-19 in relation to organ transplant recipients, including identifying risk factors, developing strategies to minimize transmission, understanding outcomes for transplant recipients who contract COVID-19, and determining the best treatment for COVID-19 in organ transplant recipients considering their immunocompromised status.

#### Topic 6: Impact of COVID-19 (n=2380, 51.2%)

##### Health Complications

From 1552 studies, we found that the long-term health complications of COVID-19 remain largely a mystery. To be more precise, there is a lack of evidence on the long-term impact of COVID-19 on the respiratory system (eg, asthma and chronic obstructive pulmonary disease), cardiovascular system (eg, hypertension, deep vein thrombosis, and pulmonary embolism), neurological system (eg, confusion, dizziness, and headache), endocrine system (eg, diabetes), hepatic system (eg, liver injury), olfactory system (loss of smell and taste), and mental health (eg, anxiety, depression, and posttraumatic stress disorder).

##### Special Groups

According to 434 studies, the impact of COVID-19 on special groups is still largely unknown. Specifically, there are still many unanswered questions related to the impact of COVID-19 on pregnancy. For example, what is its impact on the risk of preterm birth and respiratory distress? What is the long-term effect on fetuses and babies? And how is the virus transmitted from mother to baby during pregnancy, labor, or delivery? Further, there is still much to learn about the impacts of the virus on pediatric populations and patients with cancer in terms of diagnosis, treatment, and complications. The long-term effects of COVID-19 on health care workers remain uncovered.

##### Education

The COVID-19 pandemic has profoundly impacted education, with schools and universities shutting down or moving to web-based learning to slow the spread of the virus. A total of 136 studies on this subtopic revealed that there is still much unknown about the impact of COVID-19 on education.

##### Media and Communication

The COVID-19 pandemic has had a profound impact on media and communication, and while much research and analysis have been conducted, there are still many unknowns. A total of 109 studies explored this topic. From these studies, we found that there are still many unanswered questions related to media and communication. For example, Will the increased media consumption habits that have emerged during the pandemic persist in the long term, or will people revert to their prepandemic habits? How will media organizations adapt to the financial challenges posed by the pandemic, and will it lead to long-term changes in the media landscape? What will be the long-term effects of social media on the spread of misinformation and society as a whole? How will the increased reliance on digital communication tools, such as video conferencing and messaging apps, impact our relationships and social dynamics in the long term?

##### Mortality

A total of 87 studies were related to the mortality of COVID-19. According to these studies, there is a lack of evidence on factors affecting mortality rates over time and on whether new strains of the virus are more deadly or have different mortality rates than the original strain.

##### Food Security

In total, 62 studies have revealed that the impacts of COVID-19 on food security remain largely unknown. While some studies have suggested that the pandemic may worsen food insecurity in certain populations, the true extent of the problem is still unclear. In addition, the long-term effects of food insecurity during the pandemic on health and well-being are yet to be determined.

## Discussion

### Principal Findings

This study proposed an ML-based method to identify research gaps in the literature. We used COVID-19 literature as a case study. Our proposed method enabled us to identify research gaps in 21 subtopics that were grouped into 6 topics. The largest topic that was identified in more than half of the analyzed studies is the “impact of COVID-19” (topic 6). This is hardly surprising, since we, as a society, are still struggling to come to terms with the long-lasting impact of the pandemic, which continues to affect many aspects of our lives. Long-term health complications are still poorly understood, given the relatively short time since the initial outbreak, which is less than 4 years. In addition, understanding the precise impact of the disease on body systems and organs proves challenging given that it is a complex disease that affects multiple organs, there is a lack of data about the virus, and its impact may vary among different populations.

The second largest topic identified in this study is the “virus of COVID-19” (topic 1). The lack of knowledge about the COVID-19 virus is attributed to its novelty as a new strain of coronavirus not previously identified in humans. Further, tracing the origins of zoonotic diseases can be difficult as they may pass through multiple animal hosts before reaching humans. Moreover, due to its rapid evolution into new variants, studying each variation of the COVID-19 virus within a short timeframe poses a significant challenge.

Topic 3 (ie, prevention of COVID-19) was the third largest topic in this paper. The lack of knowledge in this area may be attributed to the limited long-term data on vaccine safety and efficacy. Additionally, the emergence of new variants may impact vaccine efficacy, raising concerns about the effectiveness of the current vaccines against these new variants. Further, a significant challenge in assessing the long-term effects of the COVID-19 vaccine is vaccine hesitancy, which is not fully understood and can hinder data gathering on long-term effects.

The COVID-19 pandemic has impacted health care delivery, and this was topic 5 in this study. One of the subtopics in this topic is telehealth, which has seen a significant surge. Telehealth is a relatively new technology that has only recently become widely available. As a result, there may not yet be enough data available to fully evaluate its effectiveness. Telehealth is often used in conjunction with other health care interventions, such as in-person visits or medications, which can make it difficult to separate the impact of telehealth from other factors. Importantly, there may be biases in the types of patients who are most likely to use telehealth. Thus, it is difficult to generalize findings to the broader population. Another subtopic in this topic is organ transplantation. Research gaps related to this subtopic may be attributed to the following reasons: (1) while there have been some studies on the impact of COVID-19 on organ transplant patients, the number of patients in these studies is often relatively small. These limited data can make it difficult to draw definitive conclusions about the impact of COVID-19 on this population; (2) organ transplant patients are a heterogeneous population, and the impact of COVID-19 may vary depending on factors such as age, comorbidities, and the type of organ transplant; (3) organ transplant patients may be at increased risk of complications from COVID-19 due to their underlying health conditions and the immunosuppressant drugs they take to prevent organ rejection. Therefore, it can be difficult to isolate the impact of COVID-19 from these other factors.

We found that there are research gaps related to the treatment of COVID-19 (topic 4). This may be due to several factors. First, addressing research gaps related to this topic needs many rigorous clinical trials, which usually take several years to complete all 3 phases before the licensing stage. Second, such clinical trials are very expensive, and therefore only researchers with large funds can carry out these trials.

Critical gaps in our understanding of the relationship among obesity, ethnicity, and severe COVID-19 outcomes exist (topic 2). The relationship among obesity, ethnicity, and COVID-19 outcomes is likely influenced by a complex interplay of biological, behavioral, and environmental factors, making it difficult to determine the precise nature and extent of these relationships. Disentangling the effects of these different factors on health outcomes can be challenging, and limitations in the quality and availability of data on obesity and ethnicity in certain populations can make it difficult to accurately measure the relationship between these factors. Moreover, known statistical challenges are related to controlling confounding factors such as age, sex, and comorbidities that affect the nature, behavior, and interpretation of the relationship among these factors.

In February 2020, a 2-day meeting organized by the WHO brought together more than 400 participants worldwide [28]. The objective was to develop a research road map that would facilitate and expedite global research efforts aimed at controlling the transmission of COVID-19 [28]. The research road map identified many knowledge gaps and grouped them into 8 areas. All research gaps identified in this review were mentioned in the road map except for topic 6 (impact of COVID-19). This may be attributed to the fact that this topic was less important at that stage of the pandemic (2 months after the onset of COVID-19).

After 3 months of the WHO research road map, a mixed methods study was conducted on 4087 participants (researchers, policy makers, health care workers, etc) to check which of the early WHO road map priorities are still most pressing and identify any newly emerging priorities that warrant attention [29]. The study revealed that the WHO research road map is still globally applicable. However, it identified a number of new research priorities that align with the evolving nature of the pandemic and provide insights into areas where knowledge gaps exist. One of these new research priorities is the impact of COVID-19, which aligns with topic 6 in our study.

About 10 months after the onset of COVID-19, a team from the WHO Southeast Asia Region conducted a web-based survey of 48 experts to identify COVID-19 research priorities in the Southeast Asia Region [30]. The study identified 27 research priorities, which include all 6 research topics identified in this study.

Our findings are also in agreement with previous work [31] that similarly used the CORD-19 data set to determine research priorities during the COVID-19 pandemic. The earlier study identified 10 hotspots, 4 of which overlap with our identified research gaps: virus of COVID-19, risk factors of COVID-19, prevention of COVID-19, and treatment of COVID-19. Notably, our analysis further includes 2 additional topics, which are health care delivery during COVID-19 and the impact of COVID-19. The earlier study also identified additional areas: nursing and health care, diagnosis and testing, drugs and vaccines, social psychology, infection process, and clinical characteristics. The discrepancy between the 2 sets of findings may be attributed to the period when the studies were conducted. The prior study was conducted during the initial phase of COVID-19–related research (January to September 2020), during which the emphasis was predominantly on diagnosis, testing, infection processes, and clinical characteristics. At that early stage, the short- and long-term impacts of COVID-19 were less clear, as was its effect on health care delivery.

### Limitations

This study has several limitations that need to be considered when interpreting the results. First, clusters generated by the BERTopic modeling algorithm were subject to noise. In other words, we noticed that several studies in a cluster are not relevant to that cluster. Therefore, not all studies on a topic reported a research gap related to that topic.

Second, the studies included in our analysis were limited to those published up to June 2022, given the Allen Institute for AI stopped updating the data set, and new research has been published since then. Therefore, it is likely that several research gaps identified in this review have been addressed and new research gaps have emerged.

Third, it is likely that this study missed other important research gaps for several reasons: (1) studies in the CORD-19 data set were retrieved from only 5 databases; therefore, the CORD-19 data set did not include studies from other common databases such as Scopus, Web of Science, Embase, and PsycINFO; (2) our analysis relied on abstracts rather than full texts, in which especially introduction and discussion sections commonly identify research gaps; (3) this study only considers papers in English. Consequently, potential insights from studies published in other languages may have been overlooked; and (4) we cannot rule out that we missed some terms relevant to research gaps; therefore, it is likely that many studies relevant to this work were not included in the analysis.

Fourth, we do not attempt a trend analysis in favor of a compact and concise overview, and we do not yet provide automated means to analyze original research gaps that have been addressed since the publication of a given paper. However, we believe that the presented analysis is still useful as we observe clear “hot topics” that resonate with earlier thematic research areas defined by the WHO which are extremely unlikely to have been researched fully since formulating the gap.

### Practical and Research Implications

This study’s aim is not to generate research topics automatically that, when worked on, guarantee impact or publication. Instead, we scope the landscape of research gaps outlined in the literature. This study should therefore be taken as a help to identify and prioritize “hot” topics that need addressing. This study also does not seek to replace individual literature research in a chosen topic, but it can serve as a guide to formulate specific literature search queries in specific areas related to research questions left as future work by prior publications. Therefore, literature reviews or scoping reviews are still required. Nevertheless, we anticipate that with the advent of more advanced techniques like Large Language Models, the performance of such approaches will enhance in the foreseeable future, subsequently reducing the necessity for literature reviews or scoping reviews to identify research gaps.

To overcome the above-mentioned limitations of our approach, thereby improving the identification of gaps, future research should use an up-to-date list of studies that are retrieved from the most common databases in the target area (eg, MEDLINE, Scopus, Web of Science, Embase, PsycINFO, IEEE Xplore, and ACM Digital Library). If practical, researchers should also analyze full texts, or at least discussion sections, rather than only abstracts. Moreover, additional terms related to research gaps (eg, limited evidence, inconclusive findings, and insufficient evidence) should be used to identify the relevant studies and sentences appropriate for analysis. Further, there is a need to improve the performance of the BERTopic modeling algorithm in clustering studies, specifically with respect to removing outliers from the clustering. From a technical perspective, this may require further research to combine topic modeling with statistical uncertainty quantification, such as conformal prediction [32,33].

The process of clustering text documents is an essential technique used in the text mining area, as well as in a variety of applications including ML and pattern recognition. Since clustering text documents is an optimization problem, several meta-heuristics (MH) optimization algorithms have been presented as possible solutions to this nondeterministic polynomial-time hard problem. However, while obtaining the best solution, individual optimization MH algorithms may run into serious problems including poor convergence and being stuck in local optima. To address these issues, the hybridization concept was applied to combine the strengths of 2 hybrid search methods (ie, MH algorithms) and so avoid their weaknesses.

From the dominance of topic 6, we see that most research is needed to understand the long-term effects of the pandemic. Especially where COVID-19 “temporary” solutions have become established practice (telehealth, hybrid education, the logistics of resilient food supply, etc), more research is needed to answer questions regarding the efficacy and sustainability of such solutions. Long-term health complications will continue to be a topic for quite some time in the future, given that COVID-19 has only been studied for less than 4 years. As new complications and variants emerge, this subtopic arguably has the highest potential to serve humanity and create a real impact.

### Conclusions

This paper showed that ML has the potential to identify research gaps in scientific literature. Our proposed method identified research gaps in 21 subtopics that were grouped into 6 topics: virus of COVID-19, risk factors of COVID-19, prevention of COVID-19, treatment of COVID-19, health care delivery during COVID-19, and impact of COVID-19. This study is not intended to replace individual literature research within a selected topic. Instead, it can serve as a guide to formulate precise literature search queries in specific areas associated with research questions that previous publications have earmarked for future exploration. Future research should leverage an up-to-date list of studies that are retrieved from the most common databases in the target area. When feasible, full texts or, at minimum, discussion sections should be analyzed, rather than limiting their analysis to abstracts. Moreover, additional terms related to research gaps should be used to identify the relevant studies and sentences appropriate for analysis. Furthermore, future studies could evaluate more efficient modeling algorithms, especially those combining topic modeling with statistical uncertainty quantification such as conformal prediction.

## Appendix

Multimedia Appendix 1 The code used for analysis of bibliographic data to identify research gaps.

## Abbreviations

AI: artificial intelligence
BERT: Bidirectional Encoder Representations from Transformers
CORD-19: COVID-19 Open Research Data Set
MH: meta-heuristics
ML: machine learning
TF-IDF: term frequency-inverse document frequency
WHO: World Health Organization

## Data Availability

The data that support the findings of this study are available from the corresponding author upon reasonable request.
