# Supplementary material for: Machine Learning–Based Approach for Identifying Research Gaps: COVID-19 as a Case Study
Source: JMIR Form Res. 2024 Mar 5;8:e49411. doi: 10.2196/49411 (PMC10916961; doi:10.2196/49411)
Supplement: Multimedia Appendix 1 [file formative_v8i1e49411_app1.docx]

**Appendix 1: The code used for analysis of bibliographic data to identify research gaps**

<https://github.com/hazratali/cord>
